# Bioactive Peptides from Cartilage Protein Hydrolysate of Spotless Smoothhound and Their Antioxidant Activity In Vitro

**DOI:** 10.3390/md16040100

**Published:** 2018-03-22

**Authors:** Jing Tao, Yu-Qin Zhao, Chang-Feng Chi, Bin Wang

**Affiliations:** 1 Zhejiang Provincial Engineering Technology Research Center of Marine Biomedical Products, School of Food and Pharmacy, Zhejiang Ocean University, 1st Haidanan Road, Changzhi Island, Lincheng, Zhoushan 316022, China; tj962057237@163.com (J.T.); zhaoy@hotmail.com (Y.-Q.Z.); 2 National and Provincial Joint Laboratory of Exploration and Utilization of Marine Aquatic Genetic Resources, National Engineering Research Center of Marine Facilities Aquaculture, School of Marine Science and Technology, Zhejiang Ocean University, 1st Haidanan Road, Changzhi Island, Lincheng, Zhoushan 316022, China

**Keywords:** spotless smoothhound (*Mustelus griseus*), cartilage, protein hydrolysate, peptide, antioxidant activity, antioxidant enzyme

## Abstract

In the experiment, crude proteins from spotless smoothhound (*Mustelus griseus*), cartilages were isolated by HCl-Guanidine buffer, and its hydrolysate was prepared using trypsin at pH 8.0, 40 °C with a total enzyme dose of 2.5%. Subsequently, three antioxidant peptides were purified from the hydrolysate using membrane ultrafiltration, anion-exchange chromatography, gel filtration chromatography, and reverse phase high-performance liquid chromatography. The amino acid sequences of isolated peptides were identified as Gly-Ala-Glu-Arg-Pro (MCPE-A); Gly-Glu-Arg-Glu-Ala-Asn-Val-Met (MCPE-B); and Ala-Glu-Val-Gly (MCPE-C) with molecular weights of 528.57, 905.00, and 374.40 Da, respectively, using protein amino acid sequence analyzer and mass spectrum. MCPE-A, MCPE-B and MCPE-C exhibited good scavenging activities on 2,2-diphenyl-1-picrylhydrazyl radicals (DPPH•) (EC_50_ 3.73, 1.87, and 2.30 mg/mL, respectively), hydroxyl radicals (HO•) (EC_50_ 0.25, 0.34, and 0.06 mg/mL, respectively), 2,2′-azino-bis-3-ethylbenzothiazoline-6-sulfonic acid radicals (ABTS^+^•) (EC_50_ 0.10, 0.05, and 0.07 mg/mL, respectively) and superoxide anion radicals ($O_{2}^{-}$•) (EC_50_ 0.09, 0.33, and 0.18 mg/mL, respectively). MCPE-B showed similar inhibiting ability on lipid peroxidation with butylated hydroxytoluene (BHT) in a linoleic acid model system. Furthermore, MCPE-A, MCPE-B, and MCPE-C could protect H_2_O_2_-induced HepG2 cells from oxidative stress by decreasing the content of malonaldehyde (MDA) and increasing the levels of superoxide dismutase (SOD), catalase (CAT), glutathione peroxidase (GSH-Px), and glutathione reductase (GSH-Rx). Glu, Gly, Met, and Pro in their sequences and low molecular weight could be attributed to the antioxidant activities of three isolated peptides. These results suggested that GAERP (MCPE-A), GEREANVM (MCPE-B), and AEVG (MCPE-C) from cartilage protein hydrolysate of spotless smoothhound might serve as potential antioxidants and be used in the pharmaceutical and health food industries.

## 1. Introduction

Under normal conditions, reactive oxygen species (ROS) are effectively eliminated by antioxidant defense systems, such as antioxidant enzymes and nonenzymatic factors. However, the balance between the generation and elimination of ROS is broken under pathological conditions, and uncontrolled generation of free radicals can attack proteins, membrane lipids, and DNA, which induce many health disorders including cancer, neurodegenerative, coronary heart diseases, and Alzheimer’s disease [1,2]. In addition, free radical-mediated lipid oxidation can react with proteins, amino acids, vitamins, and cholesterol during food processing, transportation and storage. The reaction will result in loss of color, nutrition, and functionality as well as undesirable off-flavors and toxic compounds [3]. There is increasing demand for antioxidants in the pharmaceutical and health food industries as well as the food processing and preservation industries. Therefore, some artificial antioxidants including butylated hydroxytoluene (BHT), butylated hydroxyanisole (BHA), and tertiary butylhydroquinone (TBHQ) show stronger antioxidant activities and have been widely applied in food preservation for retarding lipid oxidation [4,5]. However, these synthetic antioxidants might cause liver damage and carcinogenesis [5]. Therefore, there has been a major interest in searching for new, natural, and efficient antioxidants from various sources as alternatives to synthetic antioxidants.

Bioactive peptides consist of 2 to 20 amino acid residues and are inactive in the amino acid sequence of their parent proteins, and they can be released by in vitro enzymatic hydrolysis without destroying their nutritional value [3]. These low molecular weight (MW) peptides are considered to have easy absorption, high activity, and contain no hazardous immunoreactions [5,6]. Recently, seafood-derived peptides with antioxidant properties have been prepared and identified from different aquatic organisms, such as the dark muscle of tuna [7,8], skin of Alaskan pollock [9], skin and head of bluefin leatherjacket [10,11], viscera and carcass of Nile tilapia [12], gonad of jellyfish [13], monkfish muscle [14], skin of Nile tilapia [15], and pectoral fin of salmon [16]. This research indicates that seafood-derived protein hydrolysates and peptides have strong antioxidant activity and could serve as functional ingredients in food systems to protect food quality by reducing oxidative stress. In addition, bioactive protein hydrolysates and/or peptides can be applied as ingredients of functional foods due to their low cost, safety, and high nutritional and physiological value [3].

Spotless smoothhound (Mustelus griseus) belongs to Chondrichthyes Carcharhiniformes Triakidae Mustelus, mainly distributed in the Pacific Northwest. In our previous research, five antioxidant peptides including GAA, GFVG, GIISHR, ELLI, and KFPE were isolated from ethanol-soluble proteins hydrolysate of spotless smoothhound muscle, and they showed strong 2,2-diphenyl-1-picrylhydrazyl radicals (DPPH•), hydroxyl radicals (HO•), 2,2′-azino-bis-3-ethylbenzothiazoline-6-sulfonic acid radicals (ABTS^+^•), and superoxide anion radicals ($O_{2}^{-}$•) scavenging activities [17]. Acid-soluble collagen and its hydrolysate were prepared from scalloped hammerhead cartilages, and their physicochemical properties were also characterized [18,19]. However, there was no research focusing on the antioxidant peptides from spotless smoothhound cartilages. Therefore, three antioxidant peptides were prepared from trypsin-hydrolysate of spotless smoothhound cartilages, and their radical scavenging activities and inhibiting abilities on lipid peroxidation, protection on H_2_O_2_-induced HepG2 cells from oxidative stress were evaluated in this study.

## 2. Results and Discussion

### 2.1. Preparation of Protein Hydrolysate from Spotless Smoothhound Cartilage (MGCH)

The type of bioactive peptides generated from a particular protein is dependent on the primary sequence of the protein and the specificity of proteases used to generate peptides [3,20]. Proteins and peptides from cartilages of Chondrichthyes have been investigated as a good source for searching antiangiogenesis, antioxidant, and antihyperuricemic agents [21,22,23]. Therefore, in the experiment, crude proteins of spotless smoothhound cartilage were extracted using HCl-Guanidine buffer with yield of 1.69% ± 0.03% (on dry cartilage), and hydrolyzed for 4 h using trypsin at pH 8.0, 40 °C with a total enzyme dose of 2.5%. The resulted hydrolysate, named as MGCH, showed good antioxidant activity, and its radical scavenging activities on DPPH• and HO• were 63.4% ± 1.86% and 73.15% ± 2.57%, respectively, at the concentration of 20 mg protein/mL.

### 2.2. Purification of Antioxidant Peptides from MGCH

#### 2.2.1. Fractionation of MGCH Using Membrane Ultrafiltration

Protein hydrolysate is a complex mixture of active and inactive peptides with different chain length and amino acid composition. Membrane ultrafiltration is a type of separation technique for the fractionation of protein hydrolysate on their MW and enrichment concentration of peptides with specific MW ranges [3,24,25]. In the experiment, MGCH was divided into two fractions including MGCH-I (MW < 10 kDa) and MGCH-II (MW > 10 kDa) by ultrafiltration with a MW Cut Off (MWCO) membrane of 10 kDa. DPPH• and HO• scavenging activities of MGCH-I were 81.8% ± 2.68% and 89.8% ± 2.98%, respectively, at the concentration of 20 mg protein/mL, which were significantly stronger than those of MGCH (DPPH• 63.4% ± 1.86%; HO• 73.15% ± 2.57%) and MGCH-II (DPPH• 55.4% ± 3.12%; HO• 71.9% ± 1.88%) (*p* < 0.05). Li et al. [19] and Arrutia et al. [26] reported that MWs of hydrolysates play a crucial role in their functionality, and hydrolysate fractions with smaller MW showed stronger antioxidant activity than those of larger MW hydrolysates. In the text, MGCH-I with short chain peptides showed stronger radical scavenging activity, and these results were in line with previous reports that the antioxidant abilities of protein hydrolysates were negatively correlated with their average MW [3,19].

#### 2.2.2. Anion-Exchange Chromatography of MGCH-I

**Figure 1:**
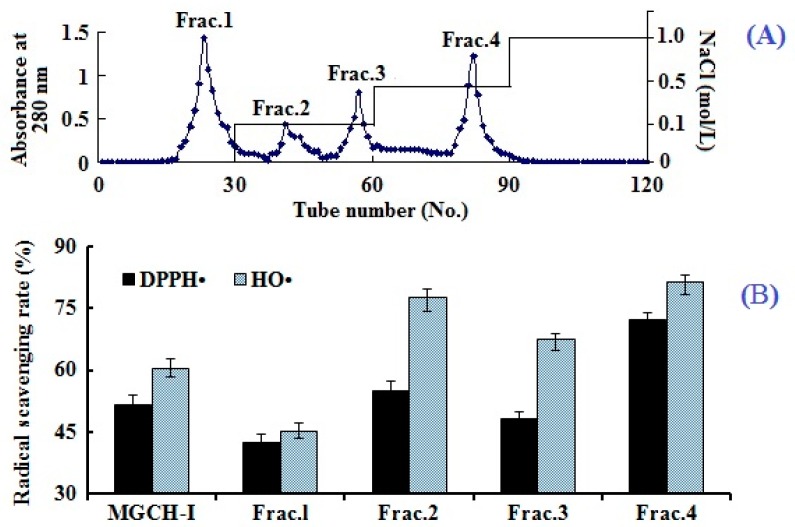
Elution profile of MGCH-I through DEAE-52 cellulose chromatography (**A**); and 2,2-diphenyl-1-picrylhydrazyl radicals (DPPH•) and hydroxyl radicals (HO•) scavenging activities of MGCH-I and its subfractions at a concentration of 15 mg protein/mL (**B**). All data are presented as the mean ± SD of triplicate results.

Ion exchange chromatography is the most popular method for purification of ions and polar molecules, such as proteins, peptides, and other charged biomolecules [27]. Separation using ion exchange chromatography depends upon the reversible adsorption of charged solute molecules to immobilize ion exchange groups of opposite charge, and the adsorption capacity was positively correlated with the charge number of isolated molecules. As shown in Figure 1A, four fractions (Frac.1 to Frac.4) were separated from MGCH-I using a DEAE-52 cellulose column. Amongst them, Frac.1 was eluted using deionized water, Frac.2 and Frac.3 were eluted using 0.1 M NaCl, and Frac.4 was eluted using 0.5 M NaCl. DPPH• and HO• scavenging activities of four prepared fractions were showed in Figure 1B, and the result indicated that Frac.4 has stronger DPPH• (72.2% ± 1.8%) and HO• (81.4% ± 3.2%) scavenging abilities than MGCH-I and other subfractions at the concentration of 15 mg protein/mL.

DEAE-52 cellulose resin is based on the diethylaminoethyl tertiary amine functional group; therefore it can interact with the molecules with negative charges and is widely used to separate proteins and peptides. Furthermore, peptides with acidic and/or hydrophobic amino acid residues such as glutamic acid (Glu), tyrosine (Tyr), methionine (Met), and leucine (Leu) are considered to have strong antioxidant activities [18,24]. Therefore, anion exchange resins including DEAE-52 cellulose and Q Sepharose FF have been used to isolate antioxidant peptides from protein hydrolysates [8,28,29]. The results indicated that the highest antioxidant activity of the peptides obtained in Frac.4 might be due to the acidic amino acid residues in their peptide sequences.

#### 2.2.3. Gel Filtration Chromatography of Frac.4

**Figure 2:**
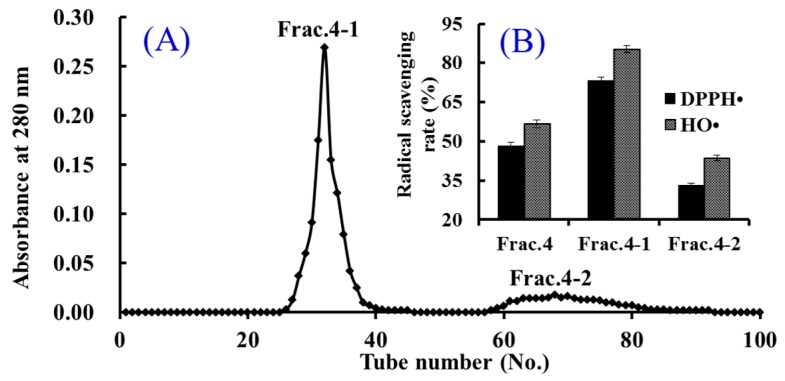
Elution profile of Frac.4 in Sephadex G-15 chromatography (**A**) and radical scavenging activities of Frac.4 and its fractions at 10 mg protein/mL concentration (**B**). All data are presented as the mean ± SD of triplicate results.

Gel filtration chromatography is an efficient technique for preparing bioactive components on the basis of molecule size [30]. Therefore, it has been widely used to analyze MW, prepare samples with particular molecule size range, and remove salt from prepared macromolecules [3,19]. In the experiment, Frac.4 was separated into two fractions of Frac.4-1 and Frac.4-2 using a Sephadex G-15 column (Figure 2A), and each fraction was collected, lyophilized, and then evaluated for DPPH• and HO• scavenging activity. As shown in Figure 2B, DPPH• and HO• scavenging activities of Frac.4-1 were 73.3% ± 1.98% and 85.4% ± 2.66%, respectively, at the concentration of 10 mg protein/mL. The radical scavenging activities of Frac.4-1 were higher than those of Frac.4 (DPPH• 48.2% ± 1.25%; HO• 56.7% ± 1.37%) and Frac.4-2 (DPPH• 33.1% ± 0.96%; HO• 43.7% ± 1.05%). In the gel filtration chromatography column, smaller molecules diffuse further into the pores of the resins and therefore move through the bed more slowly, while larger molecules enter less or not at all and thus move through the bed more quickly. Therefore, the average molecular size of Frac.4-1 was larger than that of Frac.4-2, and this result suggested that bioactivities of peptides were influenced by hydrophobicity, amino acid composition, and sequence apart from the MW [3,24].

#### 2.2.4. Isolation of Peptides from Frac.4-1 by Reverse-Phase High Performance Liquid Chromatography (RP-HPLC)

**Figure 3:**
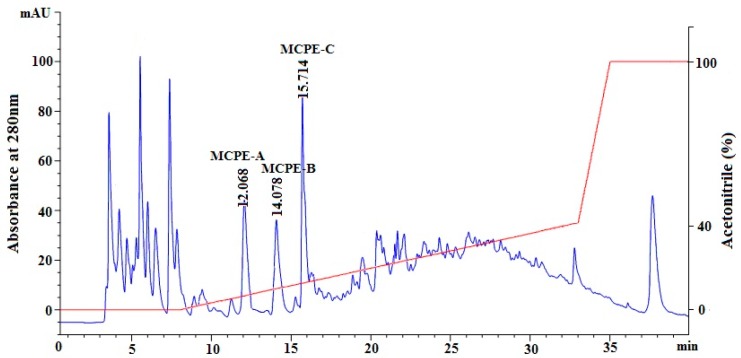
Elution profile of Frac.4-1 separated by reverse-phase high performance liquid chromatography (RP-HPLC) system on a Zorbax, SB C-18 column (4.6 × 250 mm) from 0 to 40 min.

RP-HPLC separates peptides on the basis of differences in their hydrophobicity, and the strength of the interaction between the peptides and the stationary phase depends on both hydrophobic interactions and polar interactions [5,24]. The retention time of peptides can be adjusted by increasing or decreasing organic solvent (methanol, acetonitrile) into the flowing mobile phase, and the chromatographic peak can be applied to confirm the identity and quantity of isolated peptide. Therefore, RP-HPLC has become the widespread technique for the separation of different sized peptides due to its high speed, sensitivity, and good reproducibility [24,31]. As shown in Figure 3, Frac.4-1 with the highest DPPH• and HO• scavenging activities among all hydrolysate fractions was finally measured and purified using RP-HPLC system on a Zorbax C-18 column, and the eluted fractions were collected separately according to chromatographic peaks. Among all fractions, three peptides named as MCPE-A, MCPE-B, and MCPE-C with retention times of 12.068 min, 14.078 min, and 15.714 min, respectively, showed high antioxidant activities. Therefore, MCPE-A, MCPE-B, and MCPE-C were collected and lyophilized for further research.

### 2.3. The Amino Acid Sequence Analysis and Mass Spectrometry of Peptide

**Figure 4:**
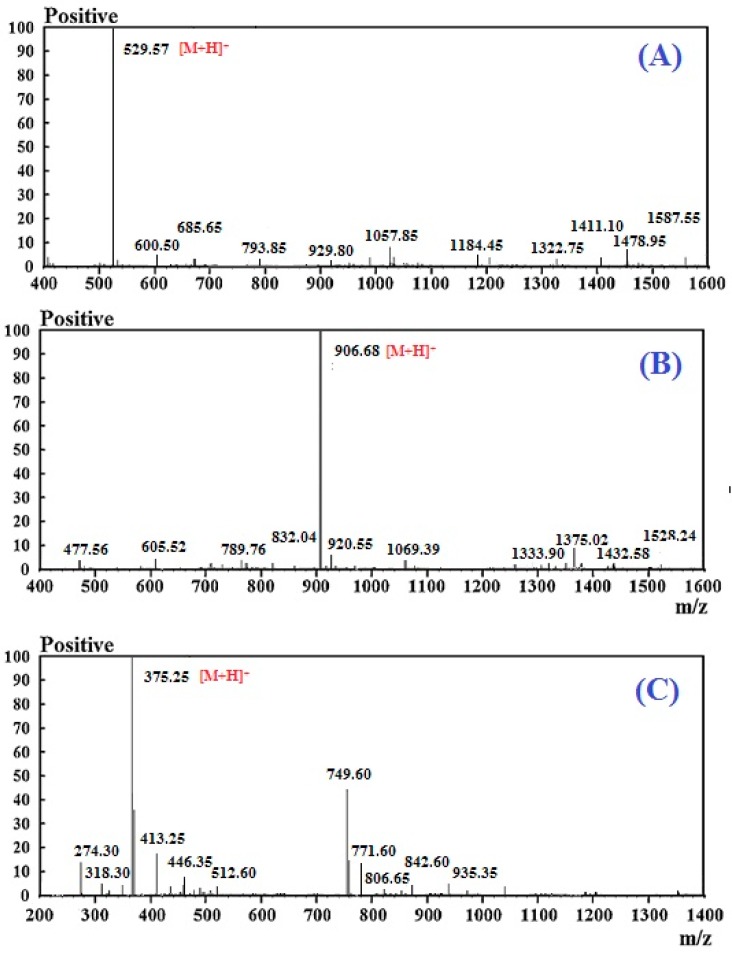
Mass spectra of Gly-Ala-Glu-Arg-Pro (MCPE-A) (**A**); Gly-Glu-Arg-Glu-Ala-Asn-Val-Met (MCPE-B) (**B**); and Ala-Glu-Val-Gly (MCPE-C) (**C**) from cartilage protein hydrolysate of spotless smoothhound.

For more detailed discussion on the structure–function relationship, the amino acid composition, sequences, and molecular mass of MCPE-A, MCPE-B, and MCPE-C were determined using protein sequencer and Q-TOF MS, and their mass spectra were shown in Figure 4. The amino acid sequences of MCPE-A, MCPE-B, and MCPE-C were identified as Gly-Ala-Glu-Arg-Pro (GAERP), Gly-Glu-Arg-Glu-Ala-Asn-Val-Met (GEREANVM), and Ala-Glu-Val-Gly (AEVG) with molecular masses of 528.57, 905.00, and 374.40 Da, respectively, which were agreed well with the theoretical masses of 528.61, 904.91, and 374.33 Da, respectively.

### 2.4. Antioxidant Activity

#### 2.4.1. DPPH• Scavenging Activity

**Figure 5:**
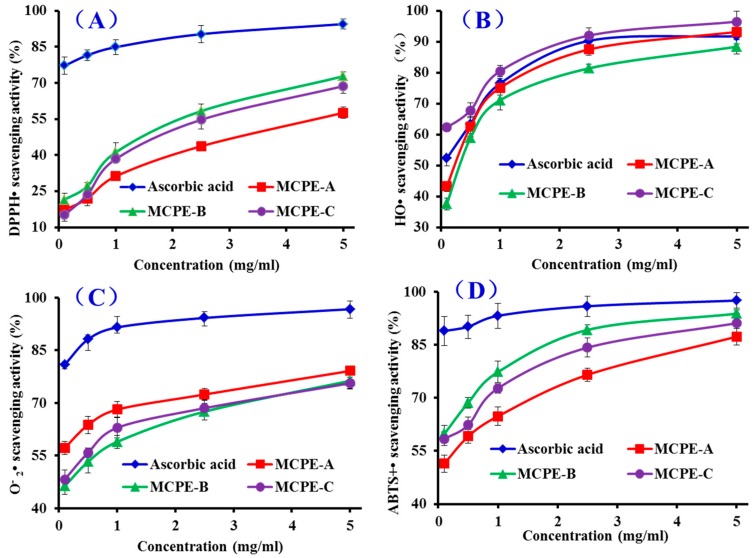
DPPH• (**A**); HO• (**B**); superoxide anion radicals ($O_{2}^{-}$•) (**C**); and 2,2′-azino-bis-3-ethylbenzothiazoline-6-sulfonic acid radicals (ABTS^+^•) (**D**) scavenging activities of MCPE-A, MCPE-B, and MCPE-C from cartilage protein hydrolysate of spotless smoothhound. All data are presented as the mean ± SD of triplicate results.

DPPH• with a single electron exhibits deep violet and shows maximal absorbance at 517 nm in ethanolic solution, and the absorbance decreases gradually while the free radicals are scavenged by accepting an electron or hydrogen radical and the color of the solution changes from deep violet to light yellow at the presence of a proton-donating substance [32]. As shown in Figure 5A, MCPE-A, MCPE-B, and MCPE-C showed moderate DPPH• scavenging activities and there was also a positive correlation between the concentration and the radical-scavenging activity. The half elimination ratio (EC_50_) values of MCPE-A, MCPE-B, and MCPE-C were 3.73, 1.87, and 2.30 mg/mL, respectively, and MCPE-B exhibited the highest radical scavenging ability among three isolated peptides, but its activity was still lower than that of the positive control of ascorbic acid at the same concentration. The EC_50_ of MCPE-B was lower than those of peptides from protein hydrolysates of loach (Pro-Ser-Tyr-Val (PSYV): 17.0 mg/mL) [33], blue mussel (Phe-Leu-Asn-Glu-Phe-Lue-His-Val (FLNEFLHV): 4.950 mg/mL) [16], bluefin leatherjacket (Trp-Glu-Gly-Pro-Lys (WEGPK): 4.438 mg/mL; Gly-Val-Pro-Leu-Thr (GVPLT): 4.541 mg/mL) [8], grass carp skin (Gly-Phe-Gly-Pro-Leu (GFGPL): 2.249 mg/mL; Val-Gly-Gly-Arg-Pro (VGGRP): 2.937 mg/mL) [34], salmon pectoral fin (Thr-Thr-Ala-Asn-Ile-Glu-Asp-Arg-Arg (TTANIEDRR): 2.503 mg/mL) [35], and skate cartilages (Phe-Ile-Met-Gly-Pro-Tyr (FIMGPY): 2.60 mg/mL; Gly-Pro-Ala-Gly-Asp-Tyr (GPAGDY): 3.48 mg/mL; Ile-Val-Ala-Gly-Pro-Gln (IVAGPQ): 3.93 mg/mL) [24]. However, the EC_50_ of MCPE-B was higher than those of peptides from protein hydrolysates of Chinese leek (Gly-Ser-Gln (GSQ): 0.61 mg/mL) [36], blue mussel (Pro-Ile-Ile-Val-Tyr-Trp-Lys (PIIVYWK): 0.713 mg/mL; Pro-Tyr-Ser-Phe-Lys (PYSFK): 1.575 mg/mL) [34], grass carp skin (His-Phe-Gly-Asp-Pro-Phe-His (HFGBPFH): 0.20 mg/mL) [37], and corn gluten meal (Phe-Leu-Pro-Phe (FLPF): 0.789 mg/mL; Leu-Pro-Phe (LPF): 0.777 mg/mL; Leu-Leu-Pro-Phe (LLPF): 1.084 mg/mL) [38]. Therefore, these results indicated that MCPE-B had the strong ability to donate an electron or hydrogen radical for inhibiting the DPPH• reaction.

#### 2.4.2. HO• Scavenging Activity

HO• is a very dangerous and highly reactive radical to the organism because it can destroy virtually all types of macromolecules including carbohydrates, nucleic acids (mutations), lipids (lipid peroxidation), and amino acids (e.g., conversion of Phenylalanine (Phe) to m-Tyrosine and o-Tyrosine). In addition, HO• only can be eliminated by endogenous and dietary antioxidants. The abilities of MCPE-A, MCPE-B, and MCPE-C were investigated, and the dose-related effects were observed at different peptide concentrations ranging from 0 to 5.0 mg/mL (Figure 5B). EC_50_ values of MCPE-A, MCPE-B, and MCPE-C were 0.25, 0.34, and 0.06 mg/mL, respectively, and MCPE-C exhibited the highest radical scavenging ability among all samples including the positive control of ascorbic acid at the same concentration. EC_50_ of MCPE-C was lower than those of peptides from protein hydrolysates of conger eel (Leu-Gly-Asn-Gly-Asp-Asp-Val-Asn (LGLNGDDVN): 0.687 mg/mL) [39], weatherfish loach (PSYV: 2.64 mg/mL) [33], mussel sauce (HFGBPFH: 0.50 mg/mL) [37], Chinese cherry seeds (Phe-Pro-Phe-Leu-Leu-Ile (FPELLI): 0.57 mg/mL; Val-Phe-ala-Ala-Leu (VFAAL): 0.31 mg/mL) [4], blue mussel (Tyr-Pro-Pro-Ala-Lys (YPPAK): 0.228 mg/mL) [40], skate cartilages (FIMGPY: 3.04 mg/mL; GPAGDY: 3.92 mg/mL; IVAGPQ: 5.03 mg/mL) [24], grass carp skin (PYSFK: 2.283 mg/mL; GFGPL: 1.612 mg/mL; VGGRP: 2.055 mg/mL) [34], and giant squid (Asn-Gly-Leu-Glu-Gly-Leu-Lys (NGLEGLK): 0.313 mg/mL; Asn-Ala-Asp-Phe-Gly-Leu-Asn-Gly-Leu-Glu-gly-Leu-Ala (NADFGLNGLEGLA): 0.612 mg/mL) [37]. MCPE-C showed strong HO• scavenging ability, which indicated that it could serve as a HO• scavenger for decreasing or eliminating the damage caused by HO• in food industries and biological systems.

#### 2.4.3. $O_{2}^{-}$• Scavenging Assay

$O_{2}^{-}$• is the most common free radical generated in vivo, and it can promote oxidative reaction to generate hydrogen peroxide and hydroxyl radical. Both $O_{2}^{-}$• and its derivatives can cause damage to DNA and membrane of cell [32]. Therefore, it is important to search safe and efficient antioxidants for scavenging $O_{2}^{-}$•. Figure 5C indicated the $O_{2}^{-}$• scavenging ratios of MCPE-A, MCPE-B, and MCPE-C drastically increased with increasing concentration ranging from 0.1 to 5 mg/mL, but their activities was still lower than that of ascorbic acid at the same concentration. EC_50_ value of MCPE-A (0.09 mg/mL) was lower than those of MCPE-B (0.33 mg/mL) and MCPE-C (0.18 mg/mL). Therefore, MCPE-A played a significant role in $O_{2}^{-}$• scavenging. EC_50_ of MCPE-A was lower than those of peptides from protein hydrolysates of mussel sauce (HFGBPFH: 0.20 mg/mL) [37], Chinese leek seeds (GSQ: 0.70 mg/mL) [36], *Mytilus coruscus* (Ser-Leu-Pro-Ile-gly-Leu-Met-Ile-Ala-Met (SLPIGLMIAM): 0.3168 mg/mL) [41], skate cartilage (FIMGPY: 1.61 mg/mL; GPAGDY: 1.66 mg/mL; IVAGPQ: 1.82 mg/mL) [24], round scad (His-Asp-His-Pro-Val-Cys (HDHPVC): 0.265 mg/mL; His-Glu-Lys-Val-Cys (HEKVC): 0.235 mg/mL) [42], and croceine croaker muscle (Tyr-Leu-Met-Arg (YLMR): 0.450 mg/mL; Val-Leu-tyr-Glu-Glu (VLYEE): 0.693 mg/mL; Met-Ile-Leu-Met-Arg (MILMR): 0.993 mg/mL) [25]. $O_{2}^{-}$• is catalyzed into hydrogen peroxide and oxygen by superoxide dismutases (SOD) in organism. Therefore, MCPE-A, MCPE-B, and MCPE-C might have similar activity with SOD to eliminate $O_{2}^{-}$• damage in biological systems.

#### 2.4.4. ABTS^+^• Scavenging Assay

ABTS^+^• scavenging assay is one of the most widely assay used to screen antiradical peptides. In this assay, the blue/green ABTS^+^• produced by oxidation of ABTS with potassium persulfate has an absorption maximum of 734 nm and can be converted back to its colorless neutral form by antioxidants following the decrease of the absorption [43]. As shown in Figure 5D, MCPE-A, MCPE-B, and MCPE-C showed strong ABTS^+^• scavenging activities in a dose-effect manner with EC_50_ values of 0.10, 0.05, and 0.07 mg/mL, respectively. MCPE-B showed the strongest ABTS^+^• scavenging activity among three isolated peptides, but still weaker than ascorbic acid at the same concentration. The EC_50_ of MCPE-B was significantly lower than those of peptides from protein hydrolysates of salmon (FLNEFLHV: 1.548 mg/mL) [16], Chinese cherry seeds (FPELLI: 0.40 mg/mL; VFAAL: 0.38 mg/mL) [4], corn gluten meal (FLPF: 1.497 mg/mL; LPF: 1.013 mg/mL; LLPF: 1.031 mg/mL) [38], skate cartilages (FIMGPY: 1.04 mg/mL; GPAGDY: 0.77 mg/mL; IVAGPQ: 1.29 mg/mL) [24], grass carp skin (GFGPL: 0.328 mg/mL; VGGRP: 0.465 mg/mL) [34], and bluefin leatherjacket heads (WEGPK: 5.407 mg/mL; Gly-Pro-Pro (GPP): 2.472 mg/mL; GVPLT: 3.124 mg/mL) [8]. These results indicated that MCPE-A, MCPE-B, and MCPE-C especially MCPE-B have the strong ability to convert ABTS^+^• to its colorless neutral form and block the free radical reaction.

#### 2.4.5. Lipid Peroxidation Inhibition Assay

**Figure 6:**
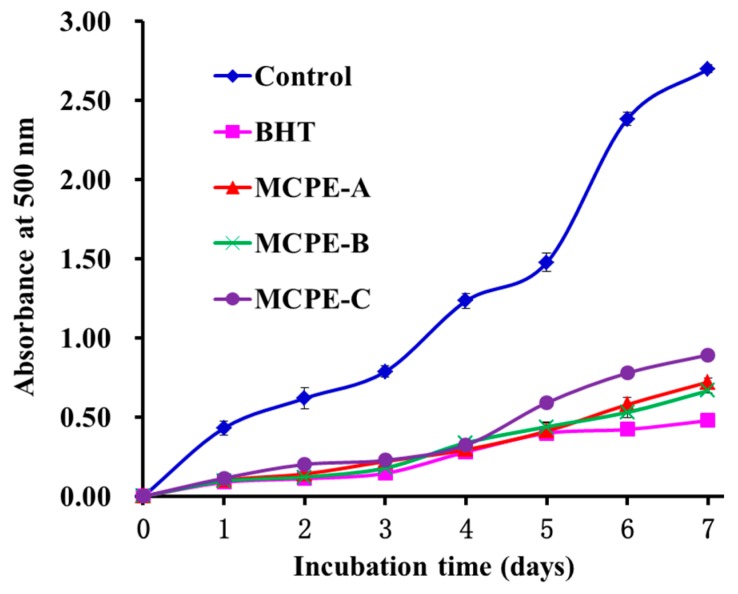
Lipid peroxidation inhibition assays of MCPE-A, MCPE-B and MCPE-C from cartilage protein hydrolysate of spotless smoothhound. All data are presented as the mean ± SD of triplicate results.

Oxidative process occurring in food or biological systems is complicated and involved in different kinds of reactions for information and propagation of lipid radicals and lipid hydroperoxides in the presence of oxygen [3,37]. In the experiment, DPPH•, HO•, ABTS^+^•, and $O_{2}^{-}$• scavenging assays had been used to assess the antioxidant activities of MCPE-A, MCPE-B, and MCPE-C, but each of these assays only measured an antioxidant property representing a different mechanism, which cannot reflect the multiple mechanisms and efficiency by which sample acted as antioxidant to retard or inhibit lipid oxidation in organism and/or food systems [3,32]. Therefore, the abilities of MCPE-A, MCPE-B, and MCPE to suppress lipid peroxidation were investigated in a linoleic acid model system. As shown in Figure 6, the 500-nm absorbance of sample solutions adding MCPE-A, MCPE-B, and MCPE-C, respectively, was significantly lower than that of the negative control (without antioxidant) and similar to that of positive control of BHT. The data indicated that three isolated peptides could effectively react with peroxyl radicals and retard lipid peroxidation in the linoleic acid emulsion system during the 7 days, and they had similar abilities on peroxidation inhibition to BHT. In addition, MCPE-A, MCPE-B, and MCPE-C are food resource-derived peptides and believed to be safer than the synthetic antioxidants, and their antioxidant activity in food system could be strengthened by increasing their using dose.

#### 2.4.6. Cytotoxicity of MCPE-A, MCPE-B, and MCPE-C in HepG2 Cells

**Figure 7:**
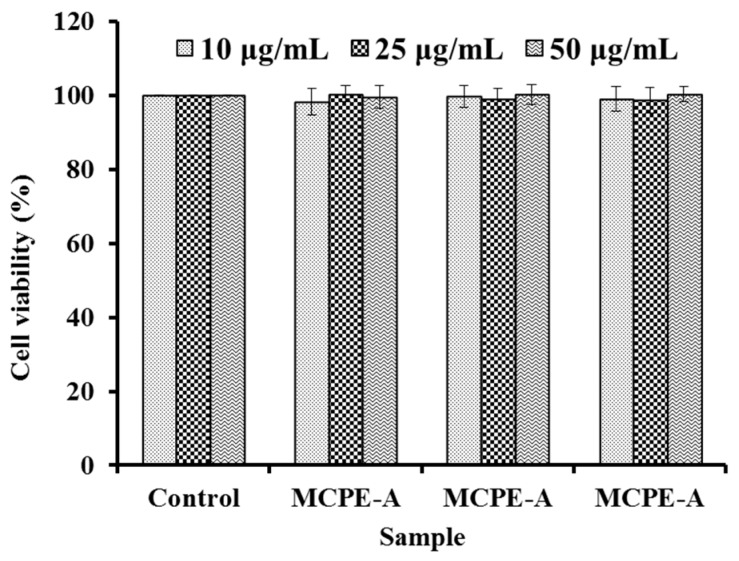
Cytotoxicity of MCPE-A, MCPE-B, and MCPE-C in HepG2 cells at concentrations of 10.0 µg/mL, 25.0 µg/mL, and 50.0 µg/mL, respectively.

Cytotoxicity of compound measured by 3-(4,5-Dimethyl-2-thiazolyl)-2,5-diphenyl-2H-tetrazolium bromide (MTT) method is an important index for drug discovery, and it can be used to screen cytotoxic compounds for developing the anticancer pharmaceuticals or compound without cytotoxic effects before investing in their development as other pharmaceuticals. Cytotoxic effects of MCPE-A, MCPE-B, and MCPE-C at the concentrations of 10.0 µg/mL, 25.0 µg/mL, and 50.0 µg/mL were determined in HepG2 cells by the MTT assay. As shown in Figure 7, MCPE-A, MCPE-B, and MCPE-C didn’t exhibited significantly cytotoxic effects on HepG2 cells compared to the control (no peptide treatment) at the tested concentrations for 24 h treatment (*p* < 0.05). The result indicated that MCPE-A, MCPE-B, and MCPE-C from cartilage protein hydrolysate of spotless smoothhound might be the candidate compounds for antioxidant drugs.

#### 2.4.7. Protection of MCPE-A, MCPE-B, and MCPE-C on H_2_O_2_-Induced Oxidative Damage HepG2 Cells

**Figure 8:**
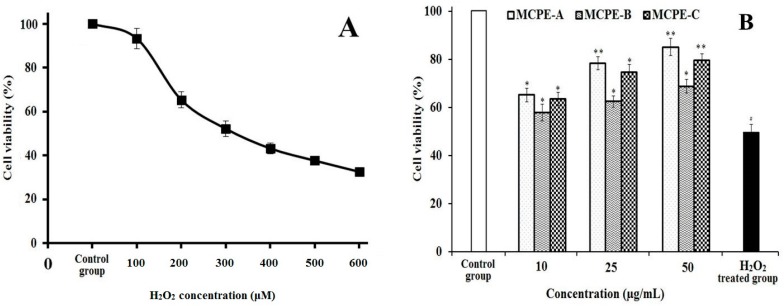
Damage effect of H_2_O_2_ on HepG2 cells (**A**) and protective effects of MCPE-A, MCPE-B, and MCPE-C on H_2_O_2_-induced oxidative damage in HepG2 cells at concentrations of 10.0 µg/mL, 25.0 µg/mL, and 50 µg/mL, respectively; (**B**) All data are presented as the mean ± SD of triplicate results. ^#^ *p* < 0.05 vs. the control group. * *p* < 0.05 vs. the H_2_O_2_ treated group. ** *p* < 0.01 vs. the H_2_O_2_ treated group.

H_2_O_2_ is a well-known powerful oxidizer and hepatotoxic chemical, and can be directly converted to hydroxyl radical and oxygen free radical. Therefore, H_2_O_2_ is often used to establish the oxidative stress model in different cells to evaluate the antioxidant activity of compounds. As shown in Figure 8A, the viability of HepG2 cells gradually reduced by H_2_O_2_ with the concentration increasing. The cell viability achieved 49.57% ± 3.21% of control group at the H_2_O_2_ concentration of 300 μM for induced 24 h. Hence, the H_2_O_2_ concentration of 300 μM was selected for the following assays of peptides oxidative stress activity evaluation. Figure 8B showed the effects of MCPE-A, MCPE-B, and MCPE-C on H_2_O_2_-induced oxidative damage HepG2 cells, and the cell viability of MCPE-A, MCPE-B, and MCPE-C treated groups were improved comparing with the H_2_O_2_ treated group without peptides. It’s worth noting that the MCPE-A treated group increased the HepG2 cell viability to 78.39% ± 2.74% and 85.14% ± 3.57% at the concentrations of 25.0 and 50.0 μg/mL, respectively, which were significantly higher than that of H_2_O_2_-induced oxidative damage group (*p* < 0.01). The present data indicated that MCPE-A, MCPE-B, and MCPE-C have strong protective effects on H_2_O_2_-induced oxidative damage HepG2 cells, especially at the high concentrations.

#### 2.4.8. Effects of MCPE-A, MCPE-B, and MCPE-C on the Antioxidant Enzymes in Oxidative Damage HepG2 Cells

Uncontrolled generation reactive oxygen species (ROS) are involved in a number of human disease states, including diabetes and cancer due to disturbance in cellular and molecular processes including cell growth, differentiation, and proliferation. Antioxidant enzymes including superoxide dismutase (SOD), catalase (CAT), glutathione peroxidase (GSH-Px), and glutathione reductase (GSH-Rx) are critical for maintaining optimal cellular and systemic health and wellbeing through stabilizing, or deactivating free radicals before they attack cellular components: SOD converts superoxide to H_2_O_2_, CAT converts H_2_O_2_ to H_2_O, GSH-Px promotes GSH elimination of H_2_O_2_, and GSH-Rx reduces glutathione disulfide (GSSG) to GSH. In addition, malonaldehyde (MDA) is the product of cell lipid oxidation which can reflect the status of oxidative stress in cells. In the present study, the effects of MCPE-A, MCPE-B, and MCPE-C on the antioxidant enzyme activities of T-SOD, CAT, GSH-Px, GSH-Rx, and content of MDA in HepG2 cells were measured for illuminating whether their protection on H_2_O_2_-induced oxidative damage HepG2 cells were associated with the modulation of endogenous antioxidant defense systems.

**Figure 9:**
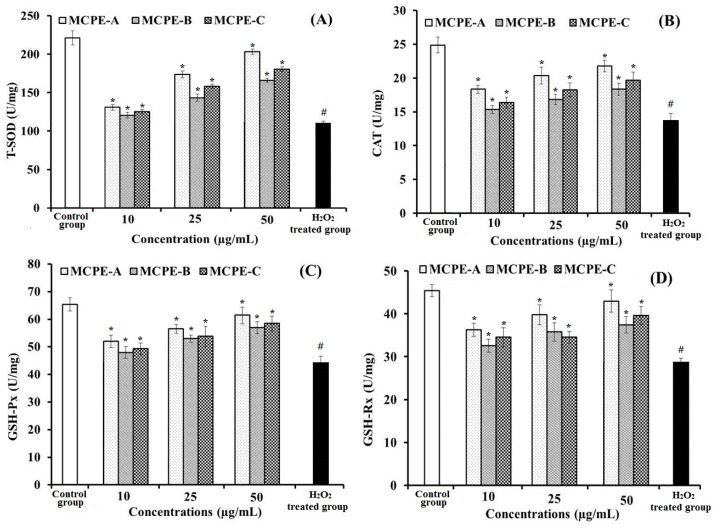

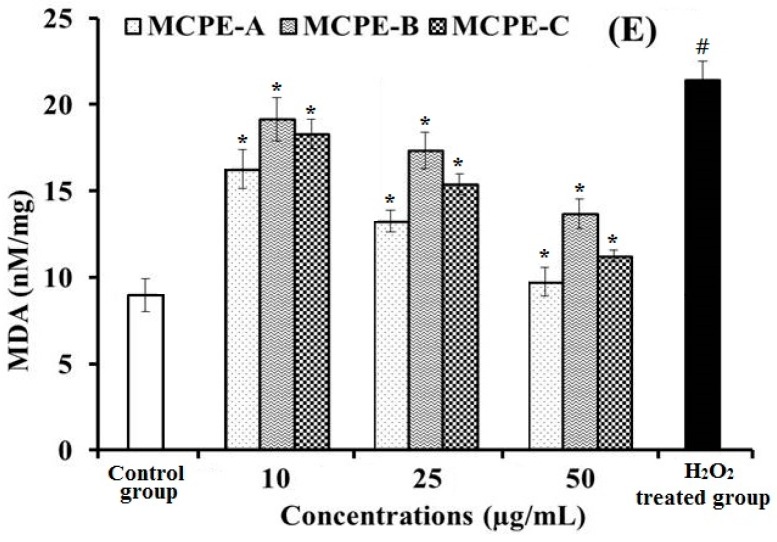
The effects of MCPE-A, MCPE-B and MCPE-C on the levels of dismutase (T-SOD) (**A**), catalase (CAT) (**B**), glutathione peroxidase (GSH-Px) (**C**), glutathione reductase (GSH-Rx) (**D**), and content of malonaldehyde (MDA) (**E**) in H_2_O_2_-induced HepG2 cells. All data are presented as the mean ± SD of triplicate results. ^#^ *p* < 0.05 vs. the control group. * *p* < 0.05 vs. the H_2_O_2_ treated group.

The levels of antioxidant enzymes (T-SOD, CAT, GSH-Px, and GSH-Rx) for all of the groups of HepG2 cells were showed in Figure 9A–D. A significant decrease in the levels of T-SOD, CAT, GSH-Px, and GSH-Rx was observed in the HepG2 cells exposed to H_2_O_2_ as compared with normal control (*p* < 0.05), and the T-SOD, CAT, GSH-Px, and GSH-Rx activities of HepG2 cells incubated by MCPE-A, MCPE-B, and MCPE-C were significantly higher than that of the H_2_O_2_ damaged group (*p* < 0.05). At the same tested concentrations, the HepG2 cell groups incubated by MCPE-A showed the highest levels of T-SOD, CAT, GSH-Px, and GSH-Rx, and followed by the groups incubated by MCPE-B. Figure 9E indicated that the MDA content (21.38 ± 1.12 U/mg prot) of damage group was significantly (*p* < 0.05) increased compared with the control group (8.96 ± 0.94 U/mg prot). The MDA contents of all protection groups were decreased by MCPE-A, MCPE-B, and MCPE-C pretreatment. At the concentration of 50.0 μg/mL, the MDA content of MCPE-A incubated group was 9.73 ± 0.82 U/mg prot, which was significantly lower than that of H_2_O_2_ treated group (*p* < 0.05). The present results indicated that MCPE-A, MCPE-B, and MCPE-C could protect H_2_O_2_-induced HepG2 cells from oxidative stress by enhancing endogenous antioxidant defense systems including the antioxidant enzyme defense system and the GSH system.

## 3. Discussion

At present, there is still lack of sufficient evidence to illuminate the relationship between the structure characteristics of peptides and their antioxidant properties to each mechanistic action. In general, molecular size, hydrophobicity, amino acid composition, and sequence are believed to play an essential role in antioxidant activity of peptides [20].

Acidic and basic amino acids play a critical role in metal ion chelating and HO• scavenging activities of peptide, which is related to carboxyl and amino groups in their side chains [3,24]. Similar results were also reported by Chang et al. who found that basic (arginine, Arg) and acidic (Glu and aspartic acid (Asp)) amino acid residues in the sequences of NTDGSTDYGILQINSR and LDEPDPLI were critical for their antioxidant activities [44]. Therefore, Glu should be one favorable factor for the antioxidant activities of MCPE-A, MCPE-B, and MCPE-C; and Arg might have positive effect on the antioxidant activities of MCPE-A and MCPE-B.

Chen et al. reported that glycine (Gly) residue might contribute significantly to antioxidant activity since the single hydrogen atom in the side chain of Gly serves as protons, and thus neutralises active free radical species [45]. Nimalaratne, Bandara, & Wu reported that the single hydrogen atom of the Gly could provide a high flexibility to the peptide backbone and positively influence the antioxidant properties [46]. Therefore, Gly residues in MCPE-A, MCPE-B, and MCPE-C might help the peptides easier to contact with the free radicals and donate proton to terminate the oxidation reaction.

Hydrophobic amino acids including proline (Pro) and Met in peptide sequences are believed to play an important role in scavenging free radicals because their large hydrophobic group can help them to facilitate the contacts with hydrophobic radical species [47]. Pyrrolidine ring of Pro could increase the flexibility of peptides and also be capable of quenching singlet oxygen due to its low ionization potential [3,48]. Chen, Muramoto, Yamauchi, & Nokihara proved that Pro played an important role in the antioxidative activity of Pro-His-His [49]. Dávalos, Miguel, Bartolomé, & López-Fandiño confirmed that tryptophan (Trp), Tyr, and Met showed the highest antioxidant activity among all the amino acids [50]. Therefore, Met residues might be the important contributor for the antioxidant activity of MCPE-B, and Pro residues might be the important contributor for the antioxidant activity of MCPE-A.

In addition, antioxidant activities of peptides highly rely on their molecular size, and shorter size peptides especially peptides with 2–10 amino acid residues are deemed to obtain stronger radical scavenging and lipid peroxidation inhibition activities than their parent native proteins and long-chain peptides [3,51]. In the test, MCPE-A, MCPE-B, and MCPE-C exhibited good antioxidant activities on radical scavenging and lipid peroxidation inhibition assays, which suggested that shorter size of MCPE-A, MCPE-B, and MCPE-C could interact more effectively and easily with free radicals and inhibit the propagation cycles of lipid peroxidation in the radical scavenging and lipid peroxidation model system [32]. But, in the experiment, MCPE-A showed the strongest $O_{2}^{-}$• scavenging activity, MCPE-B showed the strongest scavenging activity on DPPH•, ABTS^+^•, and lipid peroxidation inhibition activities, and MCPE-C exhibited the highest HO• scavenging activity among all the samples and fractions. Therefore, more detailed study should be designed for clarifying the relationship between the activities and structures of the three isolated peptides.

## 4. Experimental Section

### 4.1. Materials

Spotless smoothhound (*M. griseus*) was purchased from Nanzhen Market in Zhoushan city of China. EAE-52 cellulose and Sephadex G-15 were purchased from Shanghai Source Poly Biological Technology Co., Ltd. (Shanghai, China). Acetonitrile of LC grade and trifluoroacetic acid (TFA) were purchased from Thermo Fisher Scientific Co., Ltd. (Shanghai, China). HepG2 cells were purchased from the Cell Bank of Type Culture Collection of the Chinese Academy of Sciences (Shanghai, China). Dulbecco’s modified Eagle’s medium (DMEM), phosphate buffered saline (PBS, pH 7.2), 2,2-Diphenyl-1-picrylhydrazyl (DPPH), 2,2-azino-bis(3-ethylbenzothiazoline-6-sulfonic acid) diammonium salt (ABTS), dimethyl sulfoxide (DMSO), l-glutamine, 3-(4,5-dimethylthiazol-2-y1)-2,5-diphenyltetrazo lium bromide (MTT), and potassium bromide powder (KBr) were purchased from Sigma Chemicals Co. (Saint Louis, MO, USA). GAERP (MCPE-A), GEREANVM (MCPE-B), and AEVG (MCPE-C) with purity higher than 98% were synthesized in China Peptides Co. (Suzhou, China). All other reagents were analytical grade and purchased from Sinopharm Chemical Reagent Co., Ltd. (Shanghai, China).

### 4.2. Preparation of Protein and Its Hydrolysate from Spotless Hammerhead Cartilages

Spotless smoothhound cartilages were unfrozen, minced to homogenate and soaked in 1.0 M guanidine hydrochloride with a solid to solvent ratio of 1:5 (*w*/*v*) for 48 h, and the liquid supernatant was collected by centrifugation at 4 °C, 12,000× *g* for 10 min and dialyzed (MW 1 kDa) against 25 volumes of distilled water for 12 h with solution change every 4 h. Finally, the resulting dialysate was collected and freeze-dried.

The freeze-dried sample was dissolved (5% *w*/*v*) in 0.2 M PBS and hydrolyzed for 4 h using trypsin at pH 8.0, 40 °C with total enzyme dose 2.5%. Enzymatic hydrolysis was terminated by heating in boiling water for 10 min and the hydrolysate was centrifuged at 9000× *g* for 15 min, and the resulted supernatant named as MGCH was freeze-dried and stored at −20 °C for further analysis.

### 4.3. Isolation of Peptides from MGCH

#### 4.3.1. Fractionation of MGCH by Ultrafiltration

MGCH was fractionated using ultrafiltration (8400, Millipore, Hangzhou, China) with 10 kDa MWCO membranes (Millipore, Hangzhou, China), and two fractions termed MGCH-I (MW < 10 kDa) and MGCH-II (MW > 10 kDa) were collected and lyophilized.

#### 4.3.2. Anion-Exchange Chromatography

MGCH-I solution (5 mL, 40.0 mg/mL) was injected into a DEAE-52 cellulose column (1.6 × 80 cm) pre-equilibrated with deionized water, and stepwise eluted with 150 mL deionized water, 0.1 M NaCl, 0.5 M NaCl, and 1.0 M NaCl solution at a flow rate of 1.0 mL/min, respectively. Each eluted fraction (5 mL) was collected and measured at 280 nm. Finally, four fractions (Frac.1 to Frac.4) were pooled and lyophilized on the chromatographic peaks.

#### 4.3.3. Gel Filtration Chromatography

Frac.4 solution (5 mL, 10.0 mg/mL) was fractionated on a Sephadex G-15 column (2.6 × 160 cm) eluted with deionized water at a flow rate of 0.6 mL/min. Each elate (3 mL) was collected and monitored at 280 nm, and two fractions (Frac.4-1 and Frac.4-2) were collected and lyophilized.

#### 4.3.4. Preparative RP-HPLC

Frac.4-1 was further fractionated by a Thermo C-18 column (4.6 × 250 mm, 5 μm) (Thermo Co., Ltd., Yokohama, Japan), attached to an Agilent 1260 HPLC system (Agilent Ltd., Santa Rosa, CA, USA), and eluted with a linear gradient of acetonitrile (0–40% in 0–30 min) in 0.1% TFA at a flow rate of 0.8 mL/min. The absorbance was monitored at 280 nm. Finally, three peptides (MGCH-A, MGCH-B, and MGCH-C) were isolated and lyophilized.

### 4.4. Determination of Amino Acid Sequence and Molecular Mass

The amino acid sequences of MCPE-A, MCPE-B, and MCPE-C were determined on an Applied Biosystems 494 protein sequencer (Perkin Elmer/Applied Biosystems Inc., Foster City, CA, USA). Molecular masses were determined using a Q-TOF mass spectrometer coupled with an electrospray ionization source (ESI).

### 4.5. Antioxidant Activity

#### 4.5.1. Radical Scavenging Activities

The DPPH•, HO•, $O_{2}^{-}$•, and ABTS^+^• scavenging activities and lipid peroxidation inhibition assay were measured on the previous method [52], and the EC_50_ was defined as the concentration where a sample caused a 50% decrease of the initial concentration of DPPH•, HO•, $O_{2}^{-}$•, and ABTS^+^•, respectively.

#### 4.5.2. The HepG2 Cell Culture and Cytotoxicity Assay

The HepG2 cells were cultured in Dulbecco’s Modified Eagle’s (DMEM) medium contained 10% FBS supplemented with 2 mM L-Glu and 1% penicillin-streptomycin solution at 37 °C and 5% CO_2_ atmosphere. MTT test was used to measure the cytotoxicity of samples and the assay method was performed on the previous method [53]. After 24 h incubation in a 96-well plate (7 × 10^3^ cells/well), the HepG2 cells were cultured at the presence of different concentrations of peptide solution for 12 h. After that, the wells were washed twice with PBS and the MTT with final concentration of 0.5 mg/mL was added for an additional 4 h period. After that the formazan crystals formed by active cells were dissolved in 150 µL of DMSO. The absorbance at 570 nm was measured and the cell viability was calculated by the following equation:

Cell viability = (A_sample_/A_control_) × 100%

#### 4.5.3. H_2_O_2_-Induced Oxidative Damage and the Protection by MCPE-A, MCPE-B, and MCPE-C on HepG2 Cells

The assay was performed on the previous method described by Liang et al. [53]. The HepG2 cells were seeded on a 96-well plate with the density of 6 × 10^4^ cells/well. After culturing for 24 h, the supernatant was aspirated and H_2_O_2_ (final concentration of 100, 200, 300, 400, 500, and 600 mM) was added into the experimental group and sequentially incubated for 24 h. MTT test was applied to determine the cell viability as described in Section 4.5.2, and the optimal H_2_O_2_ concentration was confirmed when the cell viability was close to 50%.

The oxidative damaged HepG2 cells under the H_2_O_2_-induced optimal conditions were used to test the protective effects of MCPE-A, MCPE-B, and MCPE-C on the damaged cells. The peptides were dissolved in the DMEM medium with concentrations of 10.0, 25.0, and 50.0 µg/mL. The selected HepG2 cells were grown (6 × 10^4^ cells/well) in a 96-well plate for 24 h. Then the supernatant was aspirated and 100 µL of peptide samples were added into the protection groups respectively incubating for 8 h. After removing peptide samples, H_2_O_2_ was added into the damage and protection groups with the optimal concentration and sequentially incubated for optimal induced incubation time. Finally, the cell viability was measured.

#### 4.5.4. Determination of the Levels of Antioxidant Enzymes in H_2_O_2_-Induced HepG2 Cells

The assay was performed on the previous method described by Liang et al. [53]. HepG2 cells were cultured in 6-well plates (1 × 10^6^ cells/well). The isolated peptides (final concentration of 10.0, 25.0, and 50.0 µg/mL) were added into the protection groups. At last the damage and protection groups were induced by H_2_O_2_. Subsequently, 500 mL of cell lysis buffer was added into each well on ice lysed for 30 min and centrifuged at 12,000× *g*, 4 °C for 10 min. The resulted liquid supernatant was followed cold standby at 4 °C (the indicators should be measured in 6 h). The levels of T-SOD, CAT, GSHPx, GSH-Rx, and MDA were measured using assay kits according to the protocols of manufacturer. Protein concentrations were determined using the bicinchoninic acid (BCA) method to normalize the levels of T-SOD, CAT, GSH-Px, GSH-Rx, and MDA. The results were expressed as units of enzymatic activity per milligram of protein (U/mg prot).

### 4.6. Statistical Analysis

All experiments were performed in triplicate (*n* = 3), and the data are reported as the mean ± standard deviation (SD). An ANOVA test using SPSS 19.0 (Statistical Program for Social Sciences, SPSS Corporation, Chicago, IL, USA) was used to analyze the experimental data.

## 5. Conclusions

In the experiment, three peptides (MCPE-A, MCPE-B, and MCPE-C) were isolated from protein hydrolysate of spotless smoothhound (*M. griseus*) cartilage and identified as GAERP, GEREANVM, and AEVG, respectively, which all exhibited high antioxidant activities through radical scavenging and lipid peroxidation inhibition assays. In addition, MCPE-A, MCPE-B, and MCPE-C could protect H_2_O_2_-induced HepG2 cells from oxidative stress by enhancing endogenous antioxidant defense systems, including the antioxidant enzyme defense system and the GSH system. On the present results, the peptide fractions and antioxidant peptides from protein hydrolysate of spotless smoothhound cartilage may be applied as an ingredient in new functional foods, and detailed studies will be done to illustrate the relationship between the activities and structures of three isolated peptides in our lab.
